# Supplementary material for: Global hypo-methylation in a proportion of glioblastoma enriched for an astrocytic signature is associated with increased invasion and altered immune landscape
Source: eLife. 2022 Nov 22;11:e77335. doi: 10.7554/eLife.77335 (PMC9681209; doi:10.7554/eLife.77335)
Supplement: Figure 2—source data 1. [file elife-77335-fig2-data1.zip › Figure_2_source_data_1/Figure_2A/homerResults/motif81.similar.html]

motif81

## Information for motif81

C
T
A
G
G
T
C
A
A
T
C
G
G
T
A
C
C
G
A
T
A
C
T
G
C
A
G
T
A
T
G
C
A
G
T
C
A
G
T
C
C
A
T
G
A
T
C
G
C
A
T
G
A
T
C
G
C
G
A
T
A
C
T
G
A
C
T
G
T
A
C
G
A
T
C
G
C
T
G
A
  
Reverse Opposite:  

G
A
C
T
A
T
G
C
A
T
G
C
T
G
A
C
A
G
T
C
C
G
T
A
A
T
G
C
G
T
A
C
T
A
G
C
G
T
A
C
T
C
A
G
T
A
C
G
T
A
C
G
G
T
C
A
G
T
A
C
C
G
T
A
C
A
T
G
T
A
G
C
A
C
G
T
A
G
T
C
  

|  |  |
| --- | --- |
| p-value: | 1e-4 |
| log p-value: | -1.047e+01 |
| Information Content per bp: | 1.678 |
| Number of Target Sequences with motif | 13.0 |
| Percentage of Target Sequences with motif | 0.56% |
| Number of Background Sequences with motif | 5.6 |
| Percentage of Background Sequences with motif | 0.15% |
| Average Position of motif in Targets | 104.6 +/- 58.9bp |
| Average Position of motif in Background | 126.9 +/- 58.3bp |
| Strand Bias (log2 ratio + to - strand density) | 0.8 |
| Multiplicity (# of sites on avg that occur together) | 1.69 |
| Motif File: | file (matrix) reverse opposite |

### Similar de novo motifs found

|  |  |  |  |  |  |  |  |
| --- | --- | --- | --- | --- | --- | --- | --- |
| Rank | Match Score | Redundant Motif | P-value | log P-value | % of Targets | % of Background | Motif file |
